# A revised classification of Chinese Davalliaceae based on new evidence from molecular phylogenetics and morphological characteristics

**DOI:** 10.1371/journal.pone.0206345

**Published:** 2018-11-01

**Authors:** Xiao-Dong Ma, Ai-Hua Wang, Fa-Guo Wang, Chun-Mei He, Dong-Ming Liu, Pedro Gerstberger, Fu-Wu Xing

**Affiliations:** 1 Guangdong Provincial Key Laboratory of Applied Botany, South China Botanical Garden, Chinese Academy of Sciences, Guangzhou, Guangdong, China; 2 Chinese Academy of Forestry Research Institute of Forestry, Beijing, China; 3 Key Laboratory of Environment Change and Resources Use in Beibu Gulf (Guangxi Teachers Education University), Ministry of Education, Nanning, Guangxi, China; 4 Guangdong Academy of Forestry, Guangzhou, Guangdong, China; 5 Department of Plant Ecology, University of Bayreuth, Bayreuth, Bavaria, Germany; The National Orchid Conservation Center of China; The Orchid Conservation & Research Center of Shenzhen, CHINA

## Abstract

Although the phylogenetic framework of Davalliaceae is known, the classification of Chinese Davalliaceae is still controversial. In this study, a molecular phylogenetic tree of 60 accessions, including 29 species produced in China, was constructed using five plastid DNA markers—*atpB*, *atpB-rbcL*, *rbcL*, *rbcL-accD*, and *accD*. New data on studied specimens, field investigations, and scanning electron microscopy analysis of leaf epidermis and spores were used to reclassify Chinese Davalliaceae. The taxonomic position of *Davallia canariensis* was confirmed based on new evidence and a new key to sections of Chinese Davalliaceae was proposed. The taxonomically controversial genus *Paradavallodes* was confirmed as a polyphyletic group, and it was assigned to *Davallia* sect. *Trogostolon* and *Davallia* sect. *Davallodes*. Further, species endemic to China were delimited, 21 species were admitted to six sections of *Davallia*, two new combinations were proposed, two new synonyms were defined and a new key to Chinese species of Davalliaceae was presented.

## Introduction

**Fig 1:**
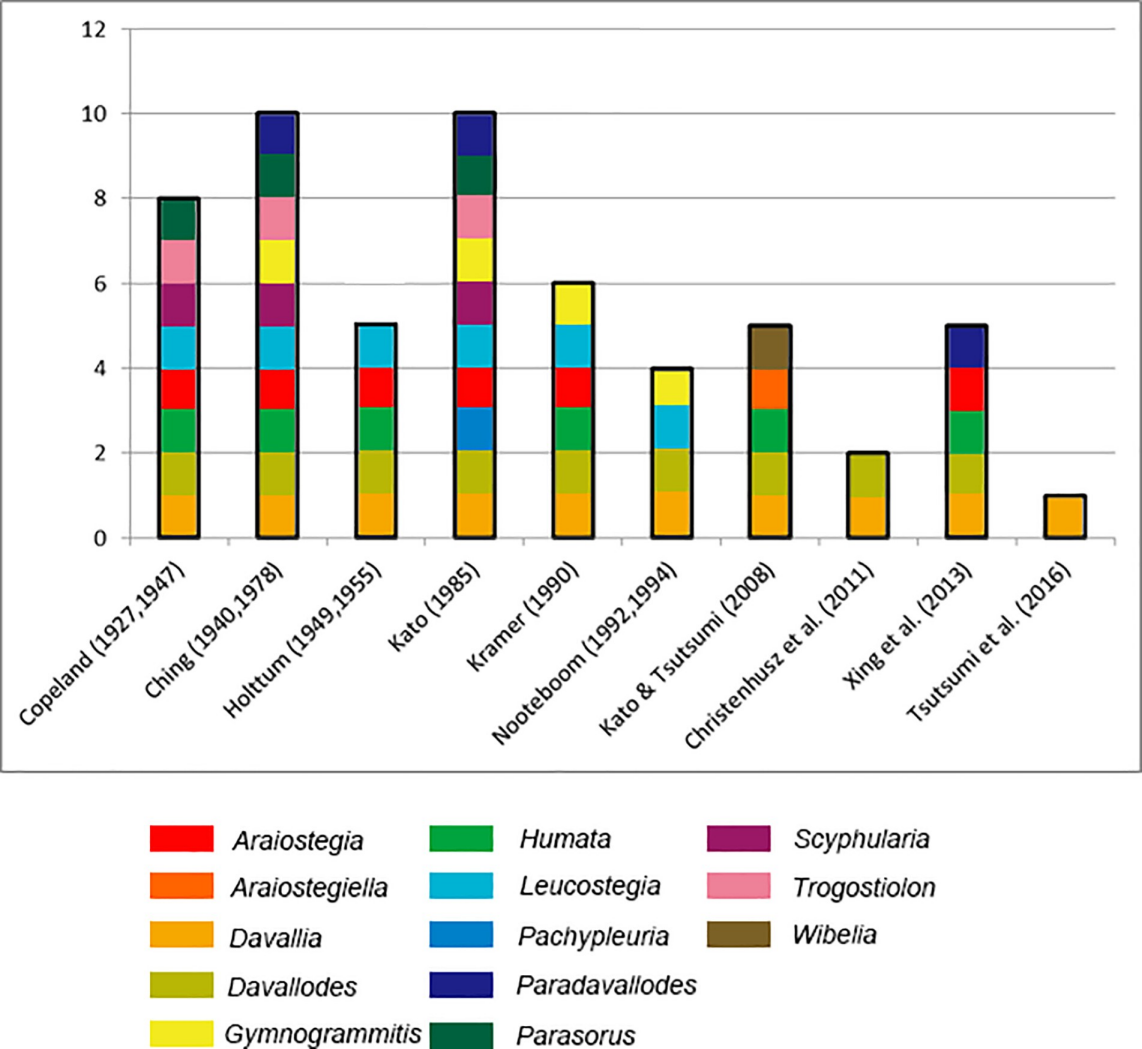
Previous generic classification of Davalliaceae. Species’ authorities and publication years are indicated in the X-axis. The Y-axis represents the total number of genera. The 13 colours represent the 13 genera reported.

Davalliaceae is a small epiphytic leptosporangiate fern family distributed in the tropics and subtropics of the Old World [1]. The circumscription and classifications of its genera have changed frequently since the establishment of this family. Previous morphology-based classifications divided Davalliaceae into 1–10 genera and approximately 49–130 species [1–21]. Classification of Davalliaceae differs significantly across different studies (Fig 1). Tsutsumi & Kato [22–23], Tsutsumi, Zhang & Kato [24], Chen [25–26], and Liu & Schneider [27] used the molecular characteristics of Davalliaceae for their classification. Tsutsumi and collaborators generated a robust phylogenetic tree of davalloid ferns by conducting a comprehensive taxonomic sampling including five combined DNA sequence datasets from plastid genes or intergenic spacers [22–24]. The phylogenetic tree obtained consisted of six main clades, and Kato & Tsutsumi [18] proposed a classification recognising the following five genera: *Araiostegiella* Kato & Tsutsumi; *Davallia* Sm.; *Davallodes* (Copel.) Copel.; *Humata* Cav.; and *Cordisquama* Bernh. Conversely, a recent study [21] revealed a phylogenetic tree containing 41 species from all the typical regions, which included seven clades. These clades were not well characterised to be distinguished at the genera level, and one genus (*Davallia*) with seven sections was proposed to classify Davalliaceae: sect. *Araiostegiella*, sect. *Davallia* (containing only *D*. *canariensis* (L.)Sm.), sect. *Davallodes* (including *Araiostegia* p.p. and *Paradavallodes*), sect. *Humata* (including *Pachypleuria* and *Parasorus*), sect. *Scyphularia* (s.l. = sect. *Davallia* sensu Kato & Tsutsumi excluding the type), sect. *Trogostolon* (s.l. = sect. *Trogostolon* sensu Kato & Tsutsumi), and sect. *Cordisquama* (s.l. = *Cordisquama* sensu Kato & Tsutsumi).

Chinese davalloid ferns are mainly distributed in the south and southwest of China; they are especially abundant in the rainforest and limestone regions of Yunnan, Guangxi, and Guangdong Provinces. However, the classification of Chinese Davalliaceae is still controversial. Ching [6, 8–10] and Ching *et al*. [7] divided Chinese Davalliaceae into nine genera, including 40 species, and published a new genus, *Paradavallodes*, which was later incorporated into *Araiostegia* by Holttum [13]. Nooteboom [17] revealed three genera with 14 species after identifying and examining Chinese Davalliaceae specimens from the major Chinese herbariums. Wu & Wang [28] revised Chinese Davalliaceae and listed five genera with 31 species (including nine species produced only in China). Xing *et al*. [20] also reviewed the classification of Chinese Davalliaceae and divided it into four genera with 17 species based on new morphological evidence. A consensus on the delimitation of Chinese Davalliaceae species is therefore difficult, especially for endemic species. For instance, *Davallia brevisora* Ching, which was believed to be an endemic species only distributed in Yunnan and Guangxi, was reconsidered as a special form of *D*. *denticulata* (Burm. f.) Mett. ex Kuhn by Nooteboom [17] or incorporated into *D*. *sinensis* (H. Christ) Ching [20]; however, other scholars [7, 28] insisted that it should remain separated as an independent endemic species. *D*. *subsolida* Ching, an endemic species of Orchid Island described by Ching *et al*. [7], which was afterwards incorporated into *D*. *solida* (G. Forst.) [20, 28], fitted the “F” morphological form of the *D*. *repens* (L.f.) Kuhn complex [26]. Other endemic species, such as *D*. *austro-sinica* Ching, *D*. *amabilis* Ching, and *Paradavallodes chingiae* (Ching) Ching were rarely collected and examined since they were described, leading to their uncertain positions. Although molecular phylogenetic analyses would be helpful for understanding the taxonomic classification positions of these species, only 14 species produced in China have been investigated by Tsutsumi & Kato [21–22] Tsutsumi *et al*. [23–24] and Chen [25–26]. An overall molecular phylogenetic study of Chinese Davalliaceae is therefore necessary to address these questions.

Recent studies [29–30] showed that the characteristics of the cuticular layer of leaf epidermis and spore ornamentation were key features for the classification of Davalliaceae at the genera/species level. The classification according to morphological traits of the leaf epidermal cuticular layer was similar to that based on the molecular phylogenetic tree presented by Tsutsumi & Kato [22–23] and Tsutsumi *et al*. [24]. Several species complexes with taxonomic controversy showed informative variations in spore ornamentation. Thus, the two morphological features are important taxonomic characters [30]. In the present study, a complementary phylogenetic tree of Davalliaceae was reconstructed by mainly focusing on species for which molecular materials were unavailable in previous studies. Further, results from field investigations, specimen examinations, observations on leaf epidermal cuticular layer and spore ornamentation, and molecular phylogenetics were integrated to obtain extensive taxonomic evidence to address the controversial taxonomic classification of Chinese Davalliaceae.

## Material and methods

### Specimen study

**Fig 2:**
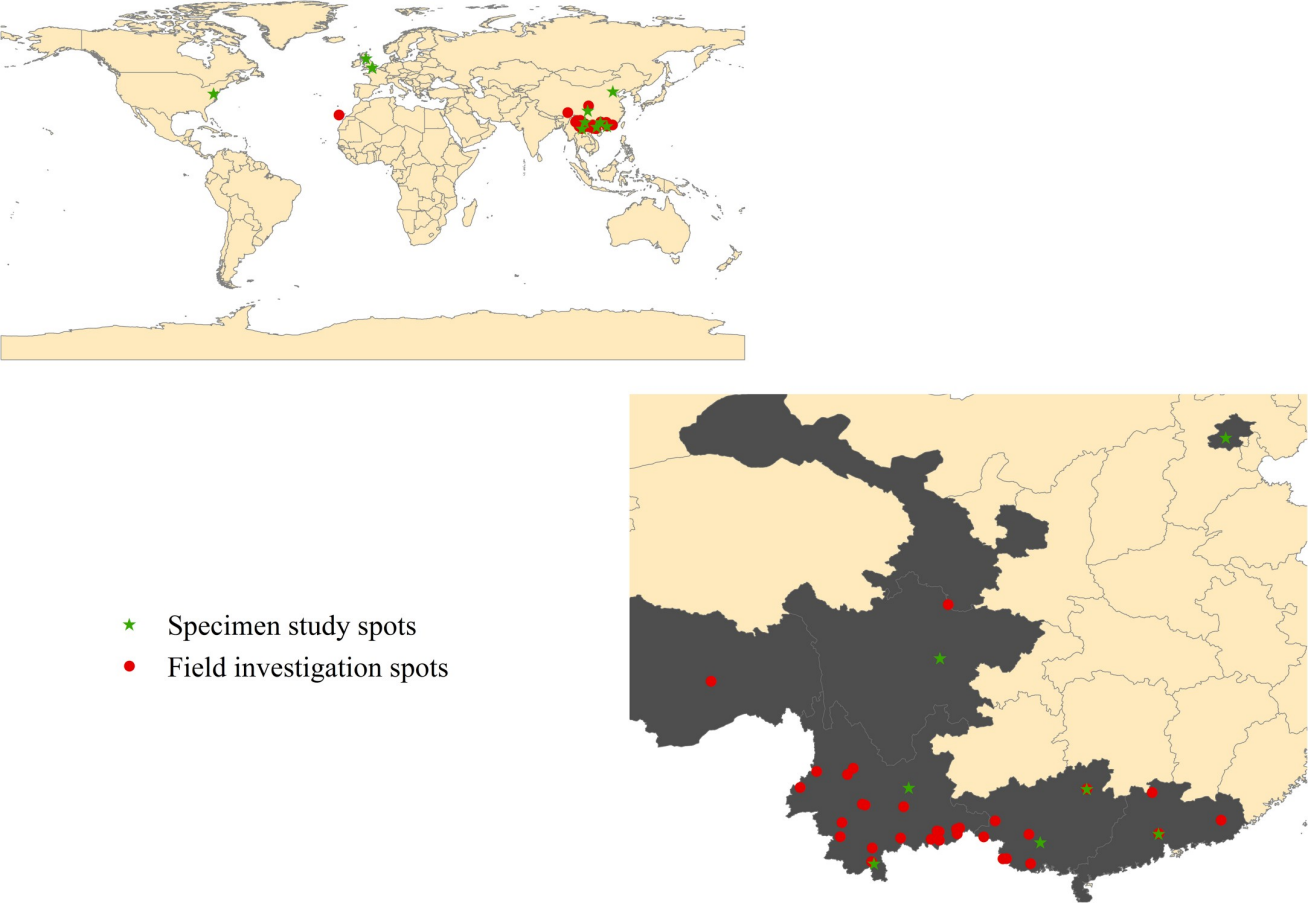
Spots of field investigation and specimen study.

We reviewed about 1700 davalloid specimens from 11 herbariums (including K, BM, E, US, PE, KUN, IBSC, HITBC, CDBI, IBK, and GXMI), and the sampling location of each specimen examined was plotted on global and China maps by using ArcGIS v. 10.1 (ESRI Inc., Redlands, CA, USA) (Fig 2). Type specimens were thoroughly examined.

### Field investigation

Field observation and sampling were widely conducted in the Canary Islands, and in Yunnan, Guangxi, Tibet, Guangdong, and Gansu Provinces of China since 2004. The geographical coordinates of sampling sites are listed in S1 Table and marked in Fig 2, and new field findings were recorded. No specific permissions were required for these locations/activities because the field studies did not involve endangered or protected species. Living plants were transplanted to the greenhouse of South China Botanical Garden, and some of them were pressed into voucher specimens, which were stored in IBSC for verification and reference.

### Molecular phylogenetic study integrated with the characters of leaf epidermal cuticular layer

About 60 accessions (including 29 species, according to the Ching [6, 8–10]and Ching *et al*. [7] taxonomic system, produced in China and two outgroups) were examined. 22 of these accessions were obtained from field sites, and the remaining 38 were downloaded from GenBank. Their localities, vouchers, and GenBank accession numbers are listed in S2 Table.

Total DNA was extracted from silica-gel-dried leaf materials by using a modified cetyl trimethylammonium bromide DNA extraction protocol [31]. Six pairs of primers, namely ‘CT-*atpB*R1 (5′-ATTGACCCTCCACTTGTAAAG-3′) and CT-spacerF (5′-ATCTATAGCTACATCTGCAAAA-3′)’, ‘CT-spacerR1 (5′-GGTGTATTATCTYTATTTGATTA-3′) and CT-*rbcL*R1 (5′-CACCAGCTTTGAATCCAAMACCTG-3′)’, ‘*rbcL*-aF (5′-ATGTCACCACAAACAGAGACTAAAGC-3′) and *rbcL*-cR (5′-GCAGCAGCTAGTTCCGGGCTCCA-3′)’, ‘CT-*rbcL*F1 (5′-ACCCAWGTCACCACAAACRGAG-3′) and CT-*rbcL*R4 (5′-CTCCACTTACTWGCWTCRCGAA-3′)’, ‘CT-*rbcL*F3 (5′-TGGCACATGCCYGCTCTAACCGA-3′) and CT-*accD*R1 (5′-CCTATACCTGTTTGAACAGCRTC-3′)’, and ‘CT-*accD*F2 (5′-ATGAARACATGACYACAAARATGT-3′) and CT-*accD*R2 (5′-ACACCTTTTAAGAGATTACGTGG-3′)’, were used to amplify the plastid gene regions *atpB*, *atpB-rbcL*, *rbcL*, *rbcL-accD*, and *accD*, respectively [22, 32]. Polymerase chain reactions (PCRs) were performed in 30 μL reaction volumes containing 0.9 μL of each primer (5p), 30–200 ng sample DNA, 0.3 U of Ex taq DNA polymerase (Takara Biomedical Technology Company, Beijing, China), 3 μL 10× buffer, 0.25 mmol L^-1^ dNTPs, and 16.9 μL ultrapure water. Amplification conditions referred to those of Hasebe *et al*. [32–33], Haufler & Ranker [34], Walsh & Sara [35], and Ebihara *et al*. [36]. The PCR products were sequenced using an ABI 3730XL platform (Majorbio Company, Shanghai, China).

Obtained were assembled in Sequencher v. 4.14, aligned using Clustal X v. 2.0 [37], and then edited manually using Bioedit v. 7.1.3 [38]. Phylogenetic trees using the sequences obtained from the combined markers (*atpB*, *atpB-rbcL*, *rbcL*, *rbcL-accD*, and *accD*) were constructed using maximum parsimony (MP) and Markov chain Monte Carlo Bayesian inference (BI). MrModeltest2 v. 2.3 [39] was used to select the general time reversible with a proportion of invariable sites and gamma distributed rate variation among sites (GTR+I+G) as the best fit molecular evolution model for the MP and BI analyses. The MP analyses were performed using PAUP*4.0b10 [40], treating gaps as missing data and using the heuristic search options with 1000 random replicates and tree-bisection-reconnection branch swapping. All characteristics were unordered and equally weighted. For BI, trees were generated for 1,000,000 generations with sampling at every 100 generations. Four chains were used with a random initial tree. For each individual data partition and for the combined dataset, the first 2500 sampled trees were discarded as burn-in to ensure that the chains reached stationarity. Nodes receiving bootstrap support *<*70% in the MP analyses or posterior probability (PP) *<*0.95 in the BI analyses were not considered well supported.

Afterwards, molecular phylogenetic analyses integrated with leaf epidermal characters (observed under scanning electron microscopy) was studied. Eight types of cuticular layer characteristics of leaf epidermis in Davalliaceae (A, Sinuate, fine, unordered stripes. B, wavy, thick, and tightly joined stripes. C, cavities visible in stripes. D, compound stripes. E, sinuolate and thick stripes, hunch shallow. F, sinuolate stripes, stripes shortened to the apophysis. G, zigzag stripes, fine stripes. H, sinuolate, fine stripes) [29] were plotted the on the present phylogenetic tree. Thirty-three accessions, for which both molecular of present study and cuticular layer of leaf epidermis data were available [29], were examined.

## Results

### Specimen study and field investigation

**Fig 3:**
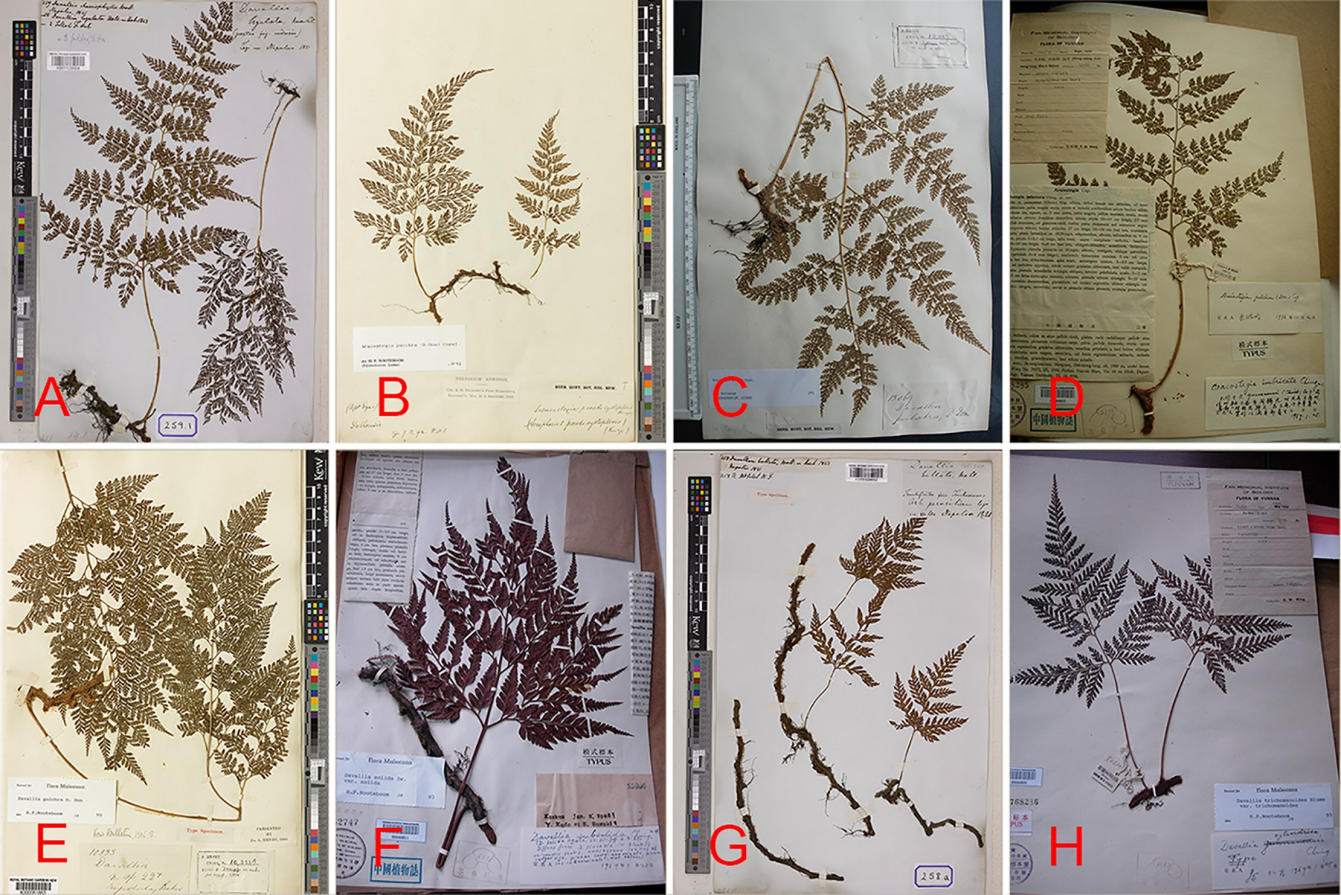
Specimens of Davalliaceae. A, *Davallia pulchra* (*Wallich259*, K). *B*, *D*. *pseudocystopteris (Colonel Dyas*, *K)*. *C*, *D*. *pulchra* (*A*. *Henry13069*, K). D, *Araiostegia imbricata* (*Wang Q*.*W*.*78372*, PE). E, *A*. *yunnanensis* (*A*. *Henry10333*, K). F, *D*. *subsolida* (*Kudo & Susuki15996*, PE). G, *D*. *bullata* (*Wallich258*, K). H, *D*. *cyclindrica* (*Wang Q*.*W*.*74303*, PE).

By intensively examining the type specimens of *Araiostegia* (= *Davallia*) *pulchra* (Don) Cop. (*Wallich259*, K, published time: 1829), *A*. (= *D*.) *pseudocystopteris* (Kunze) Cop. (*Colonel Dyas*, K, published time: 1850), *A*. (= *D*.) *beddomei* (Hope) Ching (?*71*, K, published time: 1898), *A*. *imbricata* Ching (*Wang Q*.*W*.*78372*, PE, published time: 1959), and the special *A*. (= *D*.) *pulchra* (*A*. *Henry13069*, K) (Fig 3), we confirmed that the morphological characters examined were discrepant between *A*. *pulchra* (*A*. *Henry13069*, K) and *A*. *pulchra* (*Wallich259*, K): the former has hook-shaped or oval ultimate lobes with imbricated scales densely borne on rhizome, while the latter has linear ultimate lobes with imbricated scales sparsely cling to the rhizome. According to the Chinese Virtual Herbarium (http://q.plantphoto.cn/), the type specimen photo of *A*. *pulchra* collected by Ching was not actually "*Wallich259*" but "*A*. *Henry13069*". Furthermore, we found that the right lamina of "*Wallich259*" had no distinguishing features from the lamina of "*Colonel Dyas*". In fact, *A*. *pulchra* is rather variable, and its size may differ greatly among different habitats the size may differ greatly [20]. As we observed in the wild, *A*. (= *D*.) *pseudocystopteris* is often a young stage of *A*. *pulchra*, which is the only Davalliaceae species able to grow terrestrially, and commonly presents linear ultimate lobes, imbricated scales sparsely cling to the rhizome, and rachis colour varying from pale straw to greenish. Hence, we treated *A*. (= *D*.) *pseudocystopteris* as a synonym of *A*. *pulchra*. However, *A*. *beddomei*, *A*. *yunnanensis* (Chist) Cop., and *A*. *imbricata* were not treated as synonyms of *A*. *pulchra* because they have distinct morphological characters (see list in the **Classification section**). This was corroborated by the subsequent phylogenetic analysis.

After contrasting the types of *Davallia cyclindrica* Ching (*Wang Q*.*W*.*74303*, PE, published time: 1959), *D*. *bullata* Wall. ex Hook. (*Wallich258*, K, published time: 1829), and about 10 specimens of *D*. *bullata* from K and E, we confirmed there were presence of no distinguishable features between *D*. *cyclindrica* Ching and *D*. *bullata* (Fig 3). Further, both species were distributed in the Himalayan region without geographical isolation.

*Davallia austro-sinica* and *D*. *amabilis* are both endemic Chinese species and were synchronously described by Ching *et al*. [7]. According to the description published by these authors, *D*. *austro-sinica* plant height is 30 cm and they present quadripinnate laminae, whereas *D*. *amabilis* plant height is above 100 cm and presents quadripinnate to 5-pinnate-pinnatifid laminae. To our knowledge, no studies have thoroughly compared these species since their description because of their extremely rare distributions.

During our field survey in Napo County of Guangxi Province, *Davallia amabilis* were found to be morphologically variable: the length of their mature lamina varied from 20 cm to more than 100 cm, indicating that lamina length was unstable in *D*. *amabilis*. Further, the 20-cm-thick laminae, which were similar to those of *D*. *austro-sinica*, were quadripinnate, and the 100-cm-thick laminae were quadripinnate to 5-pinnate-pinnatifid (specimens *MA057* and *MA058* in IBSC, which corresponded to different laminae of the same plant). Thus, in this case, species delimitation was difficult.

*Davallia brevisora* is an endemic Chinese species, and the delimitation between this species and *D*. *sinensis* is controversial. Nooteboom [17] regarded *D*. *brevisora* as a special form of *D*. *denticulata* and considered *D*. *sinensis* as a synonym of *D*. *solida*. Wu & Wang [28] also treated *D*. *sinensis* as a synonym of *D*. *solida*, but considered *D*. *brevisora* as a separate species. Xing *et al*. [20] treated *D*. *brevisora* as a synonym of *D*. *sinensis* but considered it distinct from *D*. *solida*. The key distinction between *D*. *sinensis* and *D*. *brevisora* is that the tubular indusium in the former is twice as long as wide, whereas the latter has cup-shaped indusium, which is slightly longer than wide or almost as long as wide [7].

*Davallia brevisora* has an extremely narrow distribution area and it is found only in Yunan and Guangxi Provinces. During the field survey in Malipo, Yunan, members of this species were found growing as epiphytes on an ancient tree. Two different forms were synchronously growing in the same plant: one with cup-shaped indusium and the other with tubular indusium (specimens *MA024* and *MA055* in IBSC, which corresponed to different laminae ofthe same plant). Therefore, the shape of the indusium is not a stable character for taxonomic delimitation. No critical circumscription is available to separate *D*. *sinensis* and *D*. *brevisora*. To confirm whether the two species are the same, we individually extracted the DNA of both lamina forms and performed phylogenetic analyses.

### Molecular phylogenetic analyses

**Fig 4:**
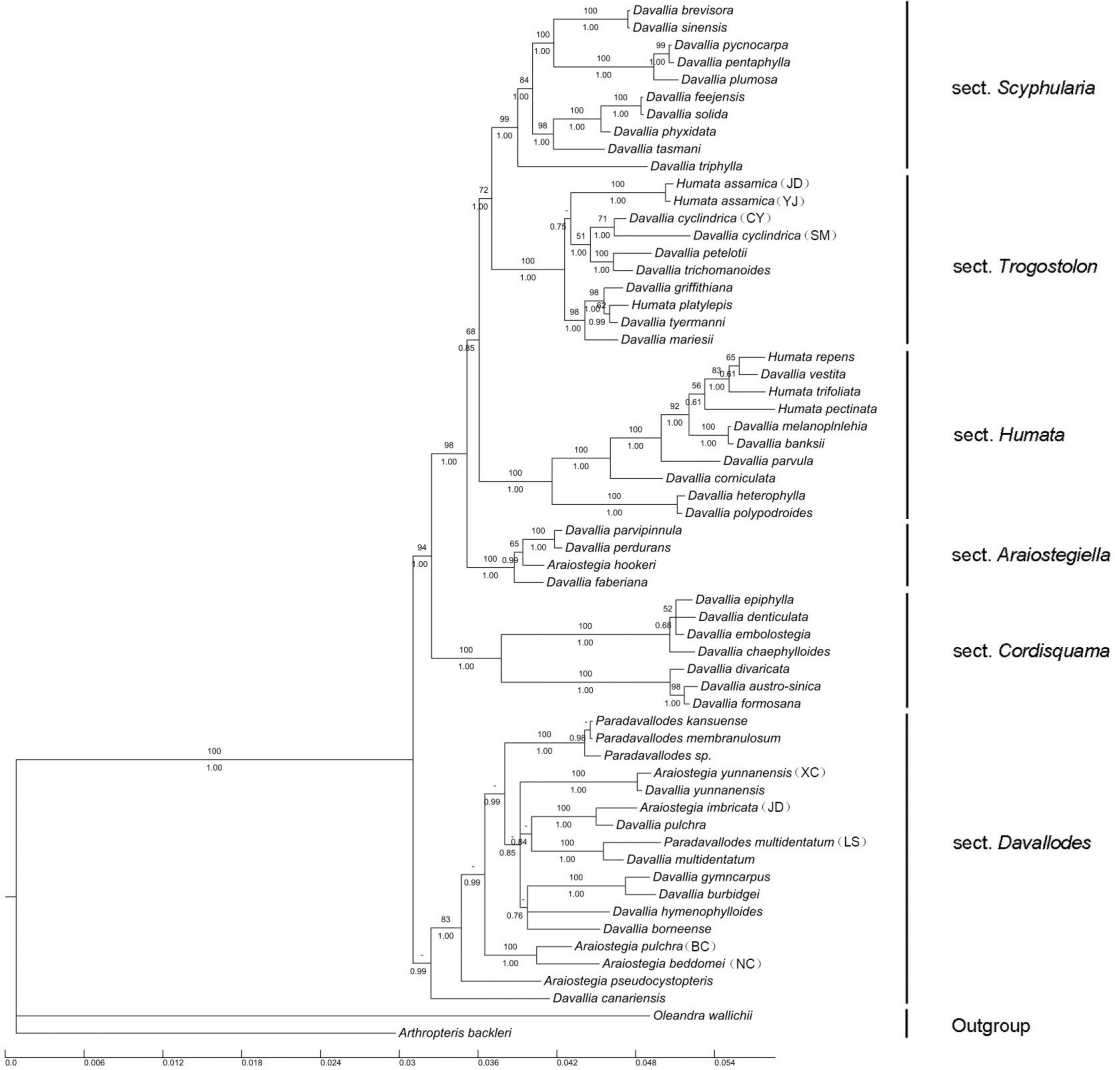
Bayesian consensus tree for Davalliaceae species, generated from *atpB*, *atpB-rbcL*, *rbcL*, *rbcL-accD*, and *accD* sequences. Bootstrap support percentages are shown above the corresponding branches, and posterior probabilities (PP) are given below the branches. Dashes indicate MP bootstrap values of less than 50%. BC, Binchuan of Yunnan; CY, Cangyuan of Yunnan; JD, Jingdong of Yunnan; LS, Lushui of Yunnan; SM, Simao of Yunnan; XC, Xichou of Yunnan; YJ, Yingjiang of Yunnan; NC, Nyingchi of Tibet.

The phylogenetic tree of Davalliaceae (with 60 accessions) generated using the five markers (*atpB*, *atpB-rbcL*, *rbcL*, *rbcL-accD*, and *accD*) comprised 5368 nucleotides, of which 1069 were variable (19.91%) and 687 were phylogenetically informative (12.80%). The MP analysis based on this dataset yielded one MP tree of 1779 steps with a consistency index of 0.6695 and a retention index of 0.8193. The tree obtained from BI analysis had a similar topology to the MP strict consensus tree (Fig 4).

The phylogenetic tree based on the new datasets (Fig 4) structurally resembled that obtained based on *atpB-rbcL-accD*, nuclear *LFY* intron 1, and *gapCp* intron [21], as it comprised six clades: (1) *Davallia* sect. *Scyphularia*; (2) *Davallia* sect. *Trogostolon*; (3) *Davallia* sect. *Humata*; (4) *Davallia* sect. *Araiostegiella*; (5) *Davallia* sect. *Cordisquama*; and (6) *Davallia* sect. *Davallodes*. The clade names followed the generic classification of Tsutsumi *et al*. [21].

### Molecular phylogenetic analyses integrated with the leaf epidermal cuticular layer characters in Davalliaceae

**Fig 5:**
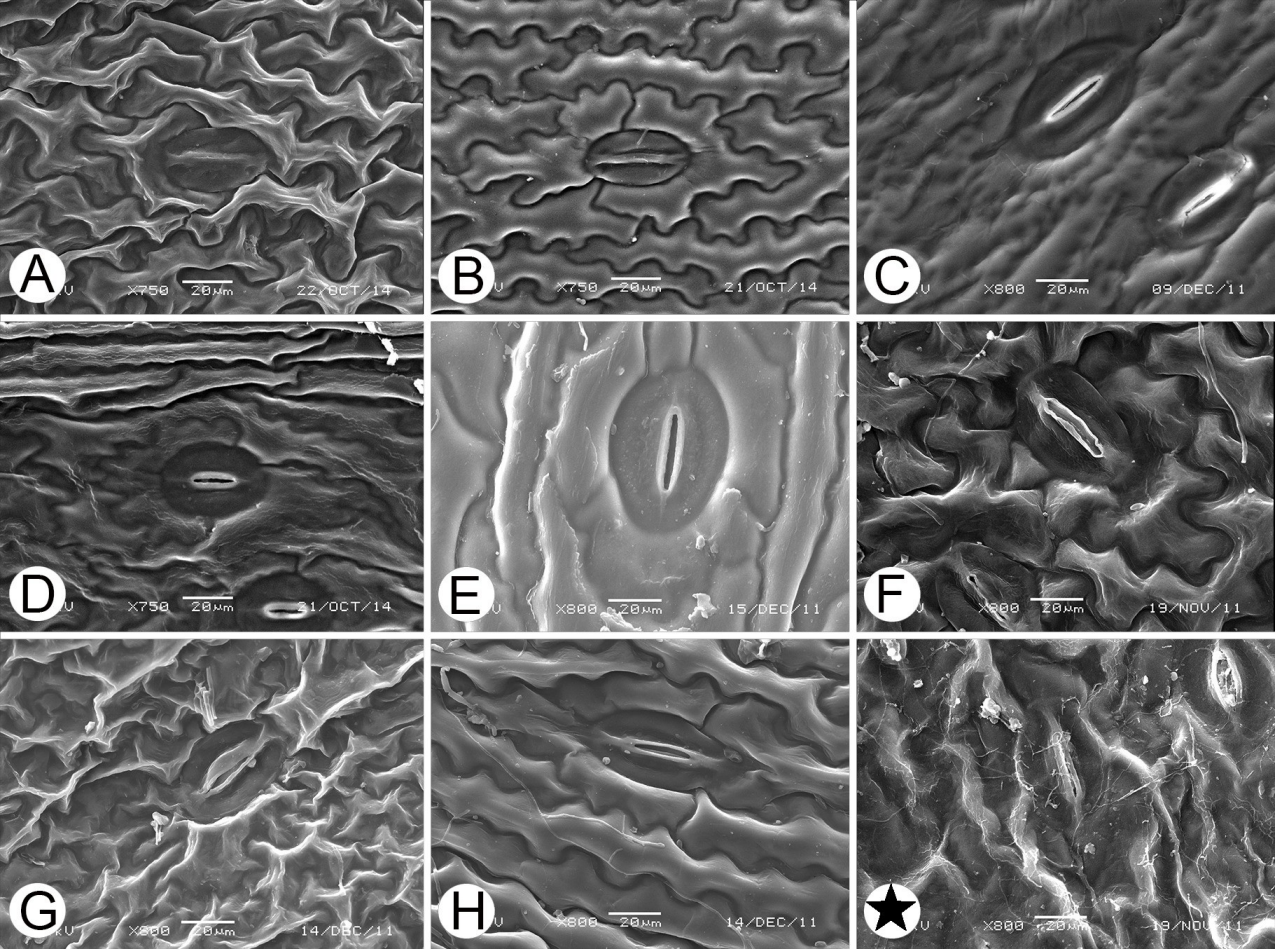
The eight types of leaf epidermis cuticular layer observed in Davalliaceae using scanning electron microscopy [29]. A, *Araiostegia beddomei*: Sinuate, fine, unordered stripes. B, *Davallia austro-sinica*: wavy, thick, and tightly joined stripes. C, *Humata pectinata*: cavities visible in stripes. D, *D*. *phyxidata*: compound stripes. E, *D*. *tyermanni*: sinuolate and thick stripes, hunch shallow. F, *Paradavallodes kansuense*: sinuolate stripes, stripes shortened to apophysis. G, *P*. *multidentatum*: zigzag stripes, fine stripes. H, *A*. *perdurans*: sinuolate, fine stripes. ★: *D*. *canariensis*.

**Fig 6:**
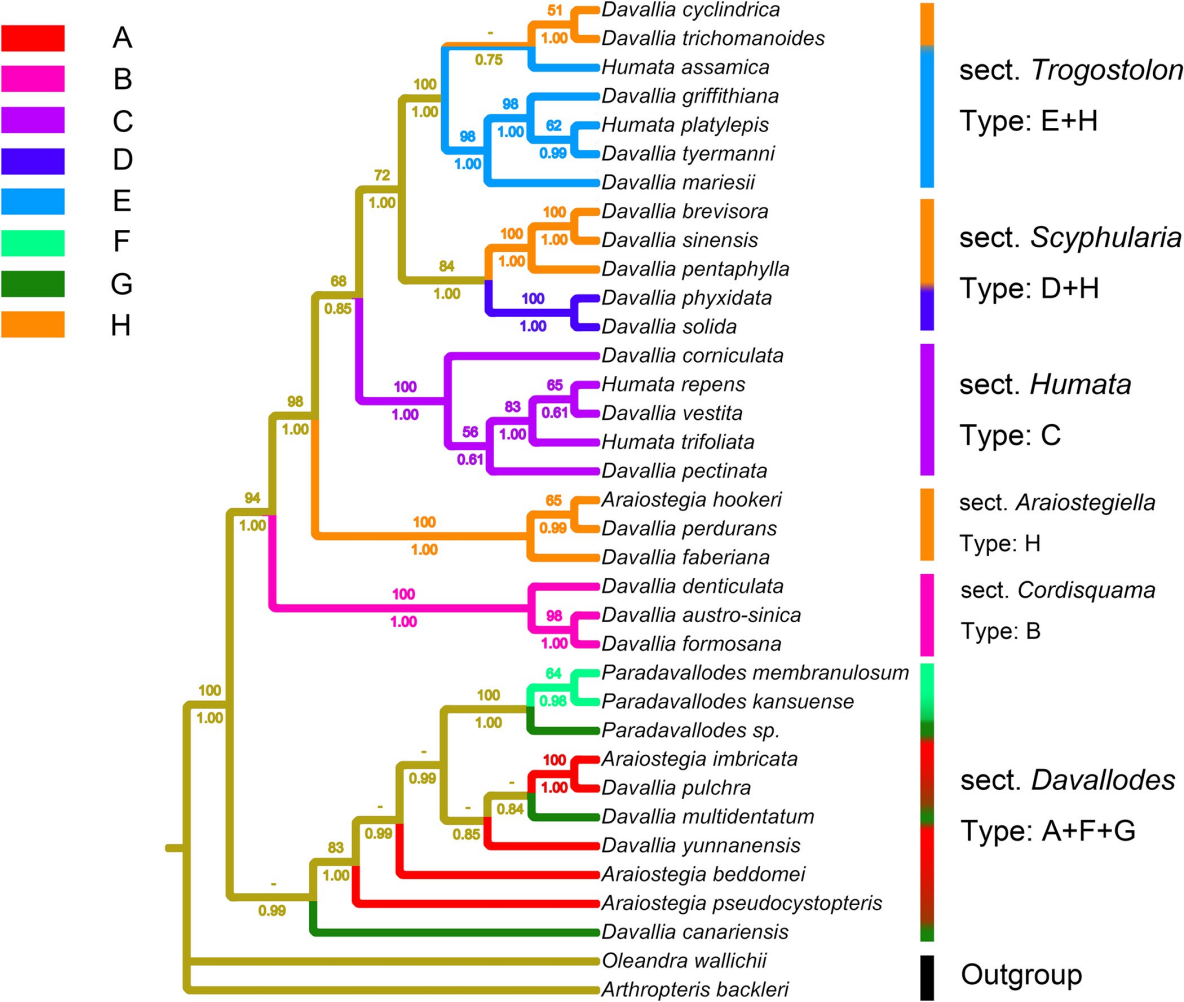
Leaf epidermal characters of Davalliaceae (observed under scanning electron microscopy) integrated with phylogenetic framework. Thirty-three accessions, for which both molecular and cuticular layer of leaf epidermis data were available, were examined. Strict consensus tree based on plastid DNA markers (*atpB*, *atpB-rbcL*, *rbcL*, *rbcL-accD*, and *accD*). Maximum parsimony (MP) bootstrap values are shown above the branches, and the Bayesian posterior probabilities are shown below the branches. Dashes indicate MP bootstrap values of less than 50%. The eight colours represent the eight types of cuticular layer characters.

Previous studies suggested that Davalliaceae can be divided into nine groups based on the cuticular layer characteristics of the leaf epidermis [29]. The examinations performed here revealed that the group represented by only *Davallia canariensis*, which was also characterised by zigzag stripes, fine stripes (Fig 5, Type G), should be included as Type G. The relationships among taxa classified according to the characteristics of leaf epidermis were unusually similar to that based on molecular phylogenies (Fig 6): The cuticular of *Davallia* sect. *Humata* has visible cavities in stripes (Figs 5 and 6, Type C), whereas that of *Davallia* sect. *Cordisquama* has wavy, thick, and tightly joined stripes (Figs 5 and 6, Type B). *Davallia* sect. *Davallodes* has sinuate (Figs 5 and 6, Type A) or zigzag (Figs 5 and 6, Type G) stripes or stripes shortened to the apophysis (Figs 5 and 6, Type F). These results indicated that the evolution of leaf epidermal cuticular layer is likely an immediate external reflection of the molecular evolution of these species. Therefore, the cuticular characteristics of leaf epidermis are important traits for the section classification of *Davallia*.

## Discussion

### Significance of our study to the taxonomy on section level of *Davallia*

Recently, Tsutsumi *et al*. [21] conducted molecular phylogenetic analysis and showed that the unstable phylogenetic position of the clade represented only by *Davallia canariensis* led to the inconsistent relationships among clades with respect to the markers analysed, and therefore the species tree derived from all datasets did not resolve the relationships. However, several close relatives of *D*. *canariensis*, such as *Araiostegia pseudocystopteris* (Kunze) Cop., *A*. *beddomei*, *Paradavallodes kansuense* Ching, and *P*. *membranulosum* (Wall. ex Hook.) Ching, were absent in the previous phylogenetic study. After these close relatives were included, phylogenetic clades with high PP were obtained andthe clade represented only by *D*. *canariensis* was merged into the clade represented by sect. *Davallodes*.In the present study, molecular data and leaf epidermis analysis results both indicated that *D*. *canariensis* is a member of *Davallia* sect. *Davallodes*. *D*. *canariensis* is generally accepted as the type of the genus *Davallia* [27]. However, if this species is classified into *Davallia* sect. *Davallodes*, the name of the section should be replaced by “*Davallia* sect. *Davallia*”. The new section classification is listed under “**Classification**”.

### *Paradavallodes* is a polyphyletic group

**Fig 7:**
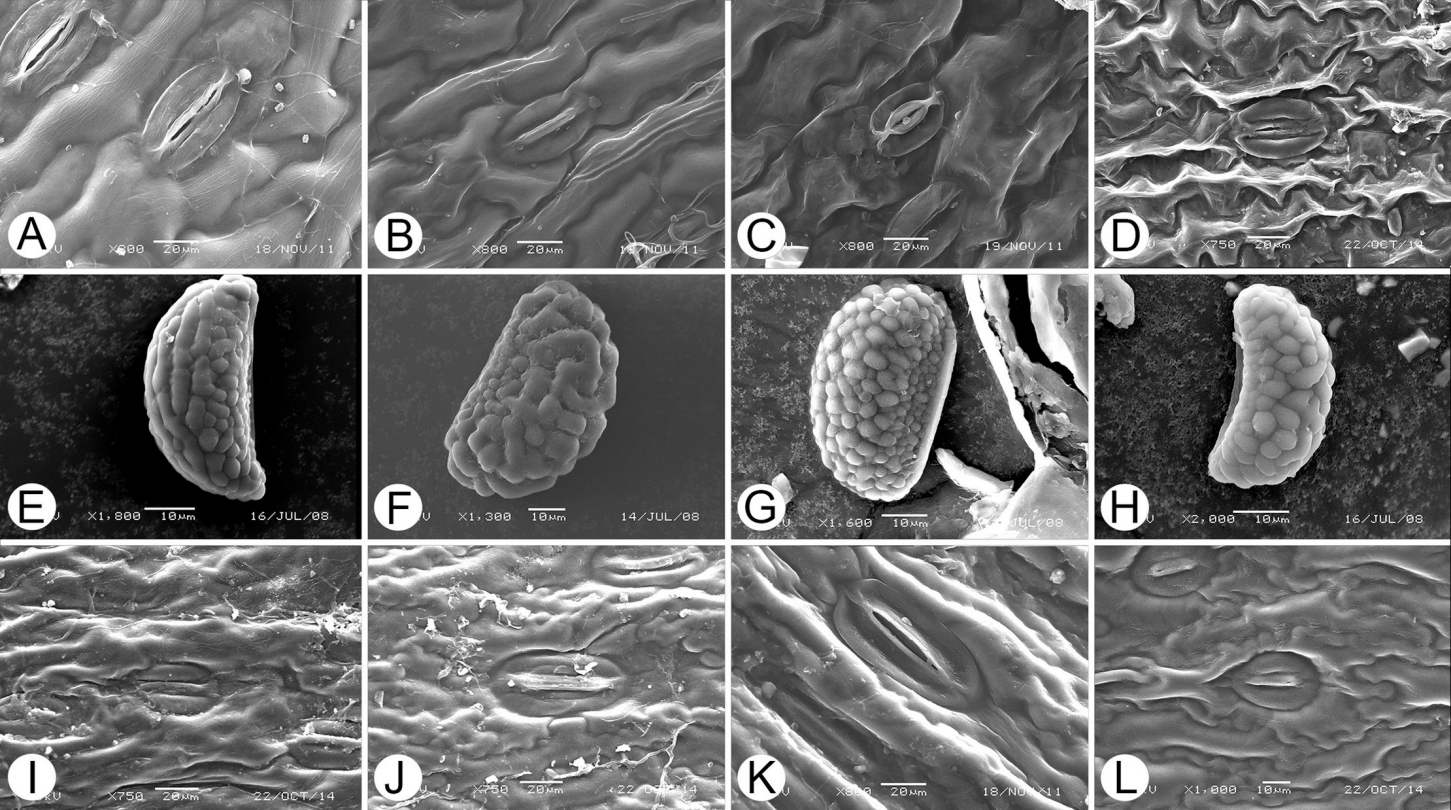
Scanning electron microscopy images [29–30]. **Leaf epidermis of:** A, *Paradavallodes chingiae* (Type E). B, *Humata assamica* (Type E). C, *P*. *membranulosum* (Type F). D, *P*. *sp*. (Type G). I, *Davallia subsolida* (Type C). J, *H*. *trifoliata* (Type C). K, *H*. *repens* (Type C). L, *D*. *solida* (Type D)**. Spores of:** E, *P*. *chingiae* (verrucate/lophate). F, *P*. *membranulosum* (verrucate fused/porous.). G, *P*. *kansuense* (verrucae colliculate). H, *P*. *multidentatum* (verrucate discrete/fused).

Genus *Paradavallodes*, comprising four species, was published by Ching [9]. Copeland [1] once divided *Paradavallodes membranulosum* (Wall. ex Hook.) Ching and *P*. *multidentatum* (Hook. et Bak.) Ching into *Davallodes* and *Araiostegia* respectively. Ching assigned these species to *Paradavallodes* based on their similar distribution (Himalayan region) and morphological characters (lamina pubescent). The examination of leaf epidermis under the scanning electronic microscope (SEM) showed that the cuticular layer of *P*. *membranulosum* and *P*. *kansuense* had sinuolate stripes, stripes shortened to the apophysis (Figs 5, 6 and 7, Type F), whereas that of *P*. *multidentatum*, a member of *Davallia* sect. *Davallodes* [21], had zigzag stripes, fine stripes (Figs 5 and 6, Type G). However, *P*. *chingiae* (Ching) Ching differed from both species by the sinuolate cuticular layer, thick stripes, and shallow hunch (Fig 7, Type E, belonging to *Davallia* sect. *Trogostolon*). The morphologically closest species to *P*. *chingiae* with similar leaf epidermis characteristics is *Humata assamica* (Bedd.) C. Chr. (Fig 7). Further, its macromorphological traits resembled those of *H*. *assamica*: both have broad lanceolate lamina, homomorphic basal and upper pinnae, and a narrow wing borne on their petiole. The spore ornamentation characters were conspicuously different between *P*. *chingiae* (verrucate/lophate) and other species of *Paradavallodes* (Figs 5 and 7). In fact, *P*. *chingiae* is the only Davalliaceae species which has lophate spore ornamentation. This indicated the particularity of the species and the necessity of it being treated as a separated species. Based on these findings, we suggest that *P*. *chingiae* belongs to *Davallia* sect. *Trogostolon* rather than *Davallia* sect. *Davallodes*.

The molecular phylogenetic study showed that *P*. *multidentatum*, *Araiostegia pulchra*, *A*. *imbricata*, *A*. *yunnanensis*, and all members of *Davallodes* (in a narrow sense) clustered into one clade. *Paradavallodes membranulosum* and *P*. *kansuense* clustered into another clade. These two clades are sister groups with high PP (0.99; Fig 4). Although *P*. *kansuense* has similar morphological characters to those of *P*. *multidentatum* [20], they are phylogenetically distant. Thus, *P*. *kansuense* should be classified as separated species rather than reserved as a synonym of *P*. *multidentatum*. All these species are nested in the *Davallia* sect. *Davallodes* (Fig 4). Integrated evidence therefore indicates that *Paradavallodes* is a polyphyletic group. *Paradavallodes multidentatum*, *P*. *membranulosum*, and *P*. *kansuense* should be assigned to *Davallia* sect. *Davallodes*, while *P*. *chingiae* should be assigned to *Davallia* sect. *Trogostolon*.

### Phylogenetic position of controversial species produced in China

#### Clarification of *A*. *pulchra* taxonomic status

The ambiguous species delimitation of *A*. *pulchra* stems from the absence of a type specimen. Although it was first published without specifying the type, Nooteboom [15] specified "*Wallich259*", and its collection time and morphological features fit well with the published time and morphological description of *A*. *pulchra*. At the same time, the author defined the broad classification concept of *A*. *pulchra* [15]: *Araiostegia beddomei*, *A*. *pulchra*, *A*. *pseudocystopteris*, *A*. *imbricata*, and *A*. *yunnanensis*, which were previously separated [7, 28, 41], were incorporated into species *A*. *pulchra*. Field surveys showed that *A*. *pulchra* (= *pseudocystopteris*) had a particular terrestrial life form, thereby differing from other members of *Araiostegia*, which required its separation. The original life form during the evolution of epiphytes in Davalliaceae and related ferns is terrestrial [23]. This is also confirmed by the position of *A*. *pulchra* (= *pseudocystopteris*), which is located at the base of the phylogenetic tree (Fig 4). According to *Flora of Tibet* [41], the purple rachis is a key character to distinguish *A*. *beddomei* from other species. However, Wu & Wang [28] selected “the density, arrangement, wrinkle of the scales”, and “the shape of ultimate pinna” rather than “purple rachis” as the key characters. Interestingly, the special individuals of *A*. *pulchra*, which fit the description of Wu & Wang [28], but grow with purple rachis, are difficult to recognise. The particular *A*. *pulchra* individual examined here was collected from Binchuan, Yunnan. The *A*. *pulchra* from Tsutsumi ‘s study [21] and *A*. *beddomei* from Tibet were also analysed in the present phylogenetic study to decipher their phylogenetic relationships among them. The endemic Chinese species *A*. *imbricata* was also examined. The phylogenetic tree (Fig 4) showed that *A*. *pulchra* from Binchuan, Yunnan and *A*. *beddomei* from Tibet clustered into a clade; *A*. *imbricata* and *A*. *pulchra* from Sikkim clustered into another clade. This suggested that the purple rachis is a key taxonomic trait in the classification of *Araiostegia*, as plants with purple rachis are closely related. The distant phylogenetic relationship between *A*. *pseudocystopteris* and *A*. *imbricata* further supported that they should be separated. Taken together with the results of previous specimen examinations, we conclude that the classification of *A*. (= *Davallodes*) *imbricata* should be reserved. The different phylogenetic positions obtained for *A*. *pulchra* (from Sikkim) and *A*. *pseudocystopteris* might be because Tsutsumi *et al*. [21] used the broad classification concept of *A*. *pulchra* that was actually unsupported by molecular data.

#### Phylogenetic position of *D*. *sinensis* (= *D*. *brevisora*)

Sequence of *Davallia sinensis* and *D*. *brevisora* were not different further supporting that *D*. *brevisora* is a synonym of *D*. *sinensis*. Molecular data (Fig 4) suggested that *D*. *sinensis*, *D*. (= *Scyphularia*) *pentaphylla* Blume, *D*. (= *Scyphularia*) *pycnocarpa* Brack., and *D*. *plumose* Baker were clustered into a clade. *D*. *sinensis* had a distant relationship with *D*. *solida* (Forst.) Sw. and *D*. *denticulata* (Burm. f.) M. Kato & Tsutsumi (*Davallia* sect. *Cordisquama*). This indicated that *D*. *sinensis* should be treated as an independent species instead of being considered synonym of *D*. *solida* or *D*. *denticulata*, and assigned to *Davallia* sect. *Scyphularia*.

#### Phylogenetic relationships among *Humata griffithiana*, *H*. *tyermanii*, *H*. *henryana*, and *H*. *platylepis*

These four species are extremely similar in morphology. Ching [7] delimited them based on the indusium shape and attached position of indusium base. According to the broad classification concept of Nooteboom [17], these species were merged into one species.

Field survey data showed that *Humata tyermanii* has become one of the key species in Guangxi Province. Notably, its indusium, similar to that of *H*. *platylepis* (Bak.) Ching, is semicircular. *Humata platylepis* is distinguished by lamina height of 35 cm, and by thick and flat rhizome. However, the shape of the indusium is not clearly different between *H*. *tyermanii* and *H*. *platylepis*. Phylogenetic data (Fig 4) showed that *H*. *griffithiana* (Hook.) C. Chr., *H*. *tyermanii*, and *H*. *platylepis* clustered into a clade. Further, the leaf epidermis and spore ornamentation characters were almost similar among the four species [29–30]. Like members of the *H*. *repens* (L. f.) Diels complex [26], *H*. *griffithiana* might undergo hybridisation and polyploidisation, leading to its high phenotypic plasticity. These results supported that *H*. *griffithiana*, *H*. *tyermanii*, *H*. *platylepis*, and *H*. *henryana* should be treated as members of the *H*. *griffithiana* complex. Considering the complexity of these species, further studies regarding their evolutionary history are needed.

#### Phylogenetic position of *D*. *cyclindrica*

According to the depiction of this Chinese endemic species [7], its architype—*Davallia bullata*—is found in K. Some scholars believed that *D*. *bullata*, *D*. *cyclindrica* Ching and *D*. *mariesii* Moore ex Baker were all synonyms of *D*. *trichomanoides* Blume [16–17, 20] and had several common characters: 3–8 mm rhizome, tripinnate lamina, flat and acicular scales. Field surveys and specimen examination confirmed *D*. *cyclindrica* a synonym of *D*. *bullata* and could be distinguished from *D*. *mariesii*. The former is characterised by the reddish brown scales of the tender stem, which is linear with subulate head, and hook-shaped or oval ultimate lobes; in contrast, the latter shows light brown or grey scales, above a considerably broader base, evenly narrowed toward the apex, and linear or sickle-shaped ultimate lobes.

The above positions are further supported by molecular data (Fig 4), showing that *D*. *cyclindrica* (= *D*. *bullata*), *D*. *petelotti* Tard. -Blot & C. Chr., and *D*. *trichomanoides* clustered into a clade. *D*. *cyclindrica* is separated from the other two species, indicating that it should be retained as a species. *Davallia mariesii* had a close relationship to *H*. *griffithiana* (variable indusia), but not to *D*. *cyclindrica*. Thus, the shape of indusia does not always reveal the evolutionary relationships of close relatives (see also *H*. *griffithiana* and *D*. *divaricata*), and therefore this character was excluded from these species identification. Thus, *D*. *cyclindrica* is a new synonym of *D*. *bullata*.

#### Phylogenetic relationships among *D*. *austro-sinica*, *D*. *formosana*, and *D*. *divaricata*

These three species are extremely similar in morphology and distinguishing them based on external morphology is difficult. The key character to distinguish them is indusium shape. In the phylogenetic tree, they clustered into a monophyletic group (Fig 4). Nooteboom [17] treated *Davallia formosana* (Hayata) M. Kato & Tsutsumi as a synonym of *D*. *divaricata* Blume. Wu & Wang [28] excluded *D*. *divaricata* from the species produced in China. After examining the specimens of *D*. *divaricata* and *D*. *formosana* in K and IBSC, we found that *D*. *divaricata* is distinguished by cup-shaped indusia, which are slightly longer than wide or almost as long and wide, whereas *D*. *formosana* has tubular indusia twice as long as wide. *D*. *austro-sinica* and *D*. *divaricata* have similar indusia. However, the phylogenetic tree (Fig 4) showed that these two species are closer to each other than to *D*. *divaricata*. This also suggests that the shape of indusia is an unstable or ambiguous classification character. Thus, *D*. *austro-sinica* and *D*. *formosana* should be treated as synonyms of *D*. *divaricata*.

#### Davallia subsolida

*Davallia subsolida* is an endemic species of Orchid Island, where *D*. *repens* is polymorphic due to polyploidy [7, 20, 26]. After examining the type specimens of *D*. *subsolida* in PE, we found that the species fitted well with the “F” morphological form of the members of the *D*. *repens* complex presented by Chen *et al*. [26] (see Fig 2 in their study). The phylogenetic position of the “F” morphological form nested in Clade X of *D*. *repens*. Furthermore, leaf epidermis SEM observations (Figs 5 and 7) showed that *D*. *subsolida* (Type C) was distinct from *D*. *solida* (Type D) and the latter is not distributed in China. Type C has a specific leaf epidermis characteristic of *Davallia* sect. *Humata* [29]. Thus, we merged *D*. *subsolida* into the *D*. *repens* complex based on geographical distribution, molecular data and leaf epidermis cuticular layer (SEM observations).

## Classification

The present study integrated the new findings from field surveys, specimens examination, the structural characteristics of leaf epidermis cuticular layer, and molecular phylogenetics, and provides a new key to identify the six section and 21 species of *Davallia* produced in China, which is presented below (Tables 1 and 2).

**Table 1:** Key to the sections of *Davallia* produced in China.

1	Lamina coriaceous, or thickly chartaceous	(2)	
+	Lamina thinly chartaceous	(5)	
2(1)	Rhizome less than 3 mm thick; scales verrucose or sometimes smooth on dorsal surface; leaf epidermis with visible cavities in stripes (SEM observation).	1	sect. ***Humata***
+	Rhizome more than 3 mm thick; scales smooth on dorsal surface; leaf epidermis with no visible cavities in stripes (SEM observation)	(3)	
3(2)	Wavy stripes in leaf epidermis, stripes thick and tightly joined (SEM observation).	2	sect. ***Cordisquama***
+	Sinuolate stripes or sometimes mingled with sinuate stripes in leaf epidermis (SEM observation)	(4)	
4(3)	Scales with long hairs extending from marginal cells.	3	sect. ***Scyphularia***
+	Scales toothed with two upturned ends of adjacent marginal cells.	4	sect. ***Trogostolon***
5(1)	Pinnae sessile; stipe and rachis persistent and turning black when pinnae fall; sinuolate stripes, stripes fine in leaf epidermis (SEM observation).	5	sect. ***Araiostegiella***
+	Pinnae of large leaves stalked; stipe and rachis not persistent; sinuate stripes or sometimes zigzag stripes, stripes fine and disorderly or shortened to apophysis in leaf epidermis (SEM observation).	6	sect. ***Davallia***

**Table 2:** Key to the species of *Davallia* produced in China.

***Davallia*** sect. ***Scyphularia——***Only *Davallia sinensis* in this section.***Davallia*** sect. ***Trogostolon——***			
1	Indusium tubular or cup-shaped	(2)	
+	Indusium circular, semicircular, or orbicular	(3)	
2(1)	Ultimate lobes linear or sickle-shaped; rhizome scales light brown, lanceolate.	1	*D*. *mariesii*
+	Ultimate lobes hook-shaped or oval; rhizome scales reddish brown or beige, linear lanceolate.	2	*D*. *bullata*
3(1)	Lamina broad lanceolate, lowest pinna similar to upper one	(4)	
+	Lamina broad ovate or triangular ovate, lowest pinna bigger than upper one.	3	*D*. *griffithiana*
4(3)	Lamina glabrous.	4	*D*. *assamica*
+	Lamina pileous.	5	*D*. chingiae*
***Davallia*** sect. ***Humata——***			
1	Lamina elongate and narrowly ovate; lowest pinna similar to upper one.	1	*D*. *pectinata*
2	Lamina ovate or triangular-ovate; lowest pinna larger than upper one	2	*D*. *repens*
***Davallia*** sect. ***Cordisquama——***			
1	False veins present.	1	*D*. *denticulata*
+	False veins absent.	2	*D*. *divaricata*
***Davallia*** sect. ***Araiostegiella——***			
1	Fronds 20–35 cm tall, proximal, 2–5 mm apart; lamina tripinnatisect, ultimate lobes long-narrow linear.	1	*D*. *hookeri*
+	Fronds 40–70 cm tall, remote, 1.5–5 cm apart; lamina 4- or 5-pinnatifid; ultimate lobes short and wide or long-narrow linear	(2)	
2(1)	Insertion between rachis and pinna with few scales, lowest pinna length about 10 cm.	2	*D*. *parvipinnula*
+	Rachis and scapes densely scaly, lowest pinna far longer	(3)	
3(2)	Basal pair of pinnules catadromous.	3	*D*. *faberiana*
+	Basal pair of pinnules opposite.	4	*D*. *perdurans*
***Davallia*** sect. ***Davallia——***			
1	Lamina pileous	(2)	
+	Lamina glabrous	(4)	
2(1)	Pinnae subsessile; rachis without scales abaxially; indusium semicircular, base rounded-truncate, persistent.	1	*D*. *membranulosum*
+	Pinnae with obvious petiole; rachis with ovate, large scales abaxially; indusium reniform, base cordate, always deciduous when mature	(3)	
3(2)	Fronds 40–60 cm height, ultimate pinna pinnatifid.	2	*D*. *multidentatum*
+	Fronds 20–40 cm height, ultimate pinna entire or few serrate at lower part.	3	*D*. kansuense*
4(2)	Rachis purple or reddish.	4	*D*. *beddomei*
+	Rachis not purple or reddish	(5)	
5(4)	Terrestrial or epiphytic in the wild, ultimate lobes linear, sparse scales cling to the rhizome.	5	*D*. *pulchra*
+	Epiphytic in the wild, ultimate lobes hook-shaped or oval, fluffy scales densely borne on rhizome	(6)	
6(5)	Lamina pentagonal ovate, lower pinna distant from the upper one, one circular scale borne on insertion between rachis and pinna.	6	*D*. *imbricata*
+	Lamina long ovate, lower pinna closely contact with the upper one, no scales borne on insertion between rachis and pinna.	7	*D*. *yunnanensis*

*: New combination.

### Taxonomy

***Davallia chingae*** (Ching) X. D. Ma & F. G. Wang., **com. nov.**

≡*Davallodes chingae* Ching in Chien et Chun, F1. Reip. Pop. Sin. 2: 283, 375. 1959; Pichi-Serm., Ind. Fil. Suppl. 4: 96. 1965.—*Paradavallodes chingae* (Ching) Ching in Acta Phytotax. Sinica 11 (1): 20. 1966; Jarrett, Ind. Fil. Suppl. 5: 126. 1985. Type: China, Yunan: K. M. Feng 13127 (holo: PE; iso: KUN).

**Distribution**: Only in Yunnan, China.

***Davallia kansuense*** (Ching) X. D. Ma & F. G. Wang., **com. nov.**

≡*Paradavallodes kansuense* Ching in Acta Phytotax. Sinica 11 (1): 20. 1966; Ching *et al*. in Fl. Tsinling. 2: 51, Pl. 12, f. 5–8. 1974; Jarrett, Ind. Fil. Suppl. 5: 126. 1985. Type: China, Gansu: Wenxian, Hsu Y. B. 1726 (holo: PE; iso: KUN).

**Distribution**: Gansu, Yunnan in China.

***Davallia bullata*** Wall. [Cat. (1829) nr. 258, nomen] ex Hook., Sp. Fil. 1845.

Type: Nepal: Wallich258 (holo:K; iso: P).

= *Davallia cylindrica* Ching **syn. nov.** in Chien et Chun, Fl. Reip. Pop. Sin. 2: 299, 375. 1959 Type: China, Yunnan: Menghai, C. W. Wang74303 (holo: PE; iso: IBSC).

***Davallia repens*** Kuhn, Fil. Deck. 27. 1867.

Type: France, Ile de France: Mascareignes, Sonnerat par Thouin (Commerson)74. (holo: P; iso: L)

= *Davallia subsolida* Ching **syn. nov.** in Chien et Chun, Fl. Reip. Pop. Sin. 2: 304, 376. 1959. Type: Taiwan: Orchid island, Kudo & Susuki15996 (PE)

## Supporting information

S1 Table Geographical coordinates of the field investigation sites. (DOCX)

S2 Table Information of the materials collected for molecular analysis and their GenBank accession numbers. (DOCX)
