# Supplementary material for: A revised classification of Chinese Davalliaceae based on new evidence from molecular phylogenetics and morphological characteristics
Source: PLoS One. 2018 Nov 1;13(11):e0206345. doi: 10.1371/journal.pone.0206345 (PMC6211685; doi:10.1371/journal.pone.0206345)
Supplement: S1 Table — (DOCX) [file pone.0206345.s001.docx]

| **Longitude** | **Latitude** | **Longitude** | **Latitude** | **Longitude** | **Latitude** |
| --- | --- | --- | --- | --- | --- |
| 104°48′35″E | 23°11′04″N | 102°31′34″E | 24°19′52″N | 106°54′12″E | 22°07′32″N |
| 104°46′35″E | 23°22′39″N | 103°42′10″E | 22°56′59″N | 106°45′17″E | 22°07′30″N |
| 104°54′34″E | 23°25′32″N | 101°09′58″E | 21°59′50″N | 106°25′16″E | 23°43′37″N |
| 103°57′11″E | 23°18′15″N | 102°24′00″E | 23°00′03″N | 105°55′59″E | 23°02′52″N |
| 104°51′11″E | 23°09′48″N | 98°08′07″E | 25°09′12″N | 113°22′29″E | 23°11′34″N |
| 104°02′18″E | 23°16′03″N | 104°02′55″E | 22°54′40″N | 116°01′21″E | 23°45′20″N |
| 100°46′46″E | 24°26′17″N | 98°50′19″E | 25°49′49″N | 113°05′31″E | 24°55′31″N |
| 100°53′23″E | 24°23′51″N | 100°09′05″E | 25°42′00″N | 104°25′26″E | 32°54′36″N |
| 101°11′55″E | 22°34′34″N | 100°23′15″E | 25°57′46″N | 94°21′44″E | 29°39′27″N |
| 99°54′37″E | 23°39′27″N | 110°18′45″E | 25°04′59″N | 16°22′45″W | 28°30′53″N |
| 99°50′40″E | 23°03′49″N | 107°54′56″E | 21°54′38″N |  |  |

**S1 Table. Geographical coordinates of the field investigation sites.**
