# Supplementary material for: A revised classification of Chinese Davalliaceae based on new evidence from molecular phylogenetics and morphological characteristics
Source: PLoS One. 2018 Nov 1;13(11):e0206345. doi: 10.1371/journal.pone.0206345 (PMC6211685; doi:10.1371/journal.pone.0206345)
Supplement: S2 Table — (DOCX) [file pone.0206345.s002.docx]

**S2 Table. Information of the materials collected for molecular analysis and their GenBank accession numbers.**

| **Species ^a^** | **Locality** | **Voucher** | **GenBank Accession No.** |
| --- | --- | --- | --- |
| ***Araiostegia*** | | | |
| *A. beddomei* (Hope) Ching | Nyingchi, Tibet, China | He001(IBSC) ^b^ | MH392510 |
| *A. hookeri* (Moore ex Bedd.) Ching | Binchuan, Yunnan, China | MA051(IBSC) | MH392501 |
| *A. imbricata* Ching | Jingdong, Yunnan, China | MA019(IBSC) | MH392506 |
| *A.* *parvipinnula* (Hayata) Cop. | Mt. Ali. Taiwan | CT1014(TI) | AB212690 |
| *A. pseudocystopteris* (Kunze) Cop. | Heqing, Yunnan, China | MA053(IBSC) | MH392511 |
| *A. pulchra* (Don) Cop. | Binchuan, Yunnan, China | MA052(IBSC) | MH392509 |
| *A. yunnanensis* (Christ) Cop. | Xichou, Yunnan, China | MA025(IBSC) | MH392508 |
| ***Davallia*** sect. ***Araiostegiella*** | | | |
| *D. faberiana* (C. Chr. ) M. Kato & Tsutsumi | China | CT1068(TI) | AB212688 |
| *D. perdurans* (Christ) M. Kato & Tsutsumi | China | CT1069(TI) | AB212691 |
| ***Davallia*** | | | |
| *D. austro-sinica* Ching | Napo, Guangxi, China | MA058(IBSC) | MH392502 |
| *D. brevisora* Ching | Malipo, Yunnan, China | MA024(IBSC) | MH392491 |
| *D. canariensis* (L.) Sm | Tenerife, Canary islands | Tenerife016(IBSC) | MH392512 |
| *D. cyclindrica* Ching | Cangyuan, Yunnan, China | MA014(IBSC) | MH392495 |
| *D. cyclindrica* Ching | Simao, Yunnan, China | MA054(IBSC) | MH392496 |
| *D. sinensis* (Christ) Ching | Malipo, Yunnan, China | MA055(IBSC) | MH392492 |
| ***Davallia*** sect. ***Scyphularia*** | | | |
| *D. fejeensis* Hook. | Fiji | CT1024(TI) | AB212703 |
| *D. pentaphylla* Blume | Java, Indonesia | CT1058(TI) | AB212723 |
| *D. plumosa* Baker | Mt. Fiamoe, Samoa. | CT1092 (TNS) | AB300576 |
| *D. pycnocarpa* Brack. | Fiji | CT1060(TI) | AB212724 |
| *D. pyxidata* Cav. | Queensland, Australia | CT1033(TI) | AB212711 |
| *D. solida* (Forst.) Sw. | New Caledonia | CT1034(TI) | AB212712 |
| *D. tasmani* Field | Univ. of Auckland, New Zealand | CT1035(TI) | AB212713 |
| *D. triphylla* Hook. | Bogor Botanic Garden, Java, Indonesia | IN113(TI) | AB212725 |
| ***Davallia*** sect. ***Trogostolon*** | | | |
| *D. griffithiana* Hook. | Taiwan | CT1026(TI) | AB212705 |
| *D. mariesii* Moore ex Baker | Gunma Pref., Japan | CT1029(TI) | AB212706 |
| *D. petelotii* Tard. -Blot & C. Chr | purchased in Laos | CT1031(TI) | AB212709 |
| *D. trichomanoides* Blume | Malaysia | CT1036(TI) | AB212714 |
| *D. tyermanii* Moore | purchased | CT1039(TI) | AB212715 |
| ***Davallia*** sect. ***Davallodes*** | | | |
| *D. borneense* (Hook.) Cop. | Poring, Sabah, Malaysia | ML94(TI) | AB212694 |
| *D. burbidgei* C. Chr. & Holttum | Mahua, Sabah, Malaysia | ML100(TI) | AB212695 |
| *D. gymnocarpum* Cop. | CT1042 | CT1042(TI) | AB212696 |
| *D.* *hymenophylloides* (Blume) M. Kato & Tsutsumi | Mt. Kinabalu, Sabah, Malaysia | CT1012(TI) | AB212689 |
| *D. multidentatum* (Hook.) M. Kato & Tsutsumi | Nyingchi–Bomi, Tibet, China | X.-C. Zhang s.n.(TNS758526, PE) | AB289538 |
| *D. pulchra* (Don) M. Kato & Tsutsumi | Sikkim, India | CT1016(TI) | AB212692 |
| *D. yunnanensis* (Christ) M. Kato & Tsutsumi | China | CT1070(TI) | AB212693 |
| ***Humata*** | | | |
| *H. assamica* (Bedd.) C. Chr*.* | Jingdong, Yunnan, China | MA015(IBSC) | MH392493 |
| *H. assamica* (Bedd.) C. Chr*.* | Yingjiang, Yunnan, China | MA041(IBSC) | MH392494 |
| *H. pectinata (Sm.) Desv.* | Orchid island, Taiwan | Wade777(IBSC) | MH392500 |
| *H. platylepis (Bak.) Ching* | Ximeng, Yunnan, China | MA040(IBSC) | MH392497 |
| *H. repens (L. f.) Diels* | Lushui, Yunnan, China | He003(IBSC) | MH392498 |
| *H. trifoliata* Cav*.* | Orchid Island, Taiwan | Wade849(IBSC) | MH392499 |
| ***Davallia*** sect. ***Humata*** | | | |
| *D. banksii* Alston | French Polynesia (Tahiti) | CT1049(TI) | AB212716 |
| *D. corniculata* (Moore) M. Kato & Tsutsumi | Cibodas, Java, Indonesia | IN30(TI) | AB212697 |
| *D. heterophylla* Desv. | Ichihara, Chiba Prefecture, Japan (cultivated. from Samoa). | CT1072 (TNS) | AB289539 |
| *D. melanophlebia* Cop. | French Polynesia (Tahiti) | CT1047(TI) | AB212717 |
| *D. parvula* (Wall. ex Hook. & Grev.) Mett. | Unknown | CT1048(TI) | AB212718 |
| *D. polypodioides* Brack. | Fiji | CT1050(TI) | AB212720 |
| *D. vestita* (Blume) Moore | Cibodas, Java, Indonesia | IN24(TI) | AB212722 |
| ***Paradavallodes*** | | | |
| *P. kansuense* Ching | Mengla, Yunnan, China | MA044(IBSC) | MH392503 |
| *P. membranulosum* (Wall. ex Hook.) Ching | Cangyuan, Yunnan, China | MA012(IBSC) | MH392504 |
| *P. multidentatum* (Hook. et Bak.) Ching | Lushui, Yunnan, China | He002(IBSC) | MH392507 |
| *P. sp.* | Cangyuan, Yunnan, China | MA002(IBSC) | MH392505 |
| ***Davallia*** sect. ***Cordisquama*** | | | |
| *D. chaerophylloides* (Poir.) Steud. | Booué, Gabon | GB-20(TNS) | AB300575 |
| *D. denticulata* (Burm. f) M. Kato & Tsutsumi | Philippines | CT1021(TI) | AB212699 |
| *D. divaricata* Blume | Irian Jaya, Indonesia | CT1020(TI) | AB212700 |
| *D. embolostegia* (Cop.) M. Kato & Tsutsumi | Sabah, Malaysia | ML56(TI) | AB212701 |
| *D. epiphylla* (G. Forst.) M. Kato & Tsutsumi | French Polynesia (Tahiti) | CT1022(TI) | AB212702 |
| *D. formosana* (Hayata) M. Kato & Tsutsumi | Taiwan | CT1025(TI) | AB212704 |
| **Outgroup** | | | |
| *Arthropteris backleri* (Hook.) Mett. | Queensland, Australia | AU308(TI) | AB212686 |
| *Oleandra wallichii* (Hook.) Presl | Chiang Mai, Thailand | TH10(TI) | AB212687 |

^a^ New accessions refer to the taxonomic systems of Ching [6, 8-10] and Ching *et al.* [7] and others refer to the classification provided by Tsutsumi *et al.* [21].

^b^ Herbarium for voucher storage.
